# Genotyping and comparative pathology of *Spirocerca* in black-backed jackals (*Canis mesomelas*) in South Africa

**DOI:** 10.1186/s12917-017-1175-4

**Published:** 2017-08-16

**Authors:** M. M. Bumby, M. C. Williams, J. C. A. Steyl, R. Harrison-White, H. Lutermann, G. T. Fosgate, P. J. de Waal, J. Mitha, S. J. Clift

**Affiliations:** 1 Section Pathology, Department of Paraclinical Sciences, Faculty of Veterinary Science, University of Pretoria, Private bag X04, Onderstepoort, 0110 South Africa; 2 Wildlife Damage- Research and Management, North West Parks and Tourism Board, Madikwe and SA Lombard Nature Reserves, North West province, South Africa, P.O. Box 783540, Sandton, Johannesburg, 2146 South Africa; 3 Mammal Research Institute, Department of Zoology and Entomology, University of Pretoria, Private Bag X20, Hatfield, Pretoria, 0028 South Africa; 4 Department of Production Animal Studies, Faculty of Veterinary Science, University of Pretoria, Private bag X04, Onderstepoort, 0110 South Africa; 5 Department of Genetics, University of Pretoria, Pretoria, 0002 South Africa

**Keywords:** Aortic aneurysms, Black-backed jackal, Genotyping, Esophageal nodule, Resistance, *Spirocerca lupi*, Spirocercosis

## Abstract

**Background:**

The pathology of spirocercosis, a disease caused by the infestation of carnivores with the nematode *Spirocerca lupi,* has been extensively described in domestic dogs and coyotes. However, it has not been described in wild carnivores in South Africa. The aim of this study was to evaluate whether black-backed jackals are a host for *Spirocerca* species and to provide a detailed description of the associated pathology. Jackals were also stratified according to age and the *Spirocerca* species recovered were characterized using molecular techniques.

**Methods:**

Standard necropsies were performed on routinely culled jackals from three of the nine provinces of South Africa during the period June 2012 to February 2013. Jackals were screened for the presence of pathognomonic *Spirocerca-*induced lesions and for evidence of aberrant migration. Relevant samples were submitted for histopathology and collected larvae were genotyped at nine microsatellite loci.

**Results:**

*Spirocerca lupi*-associated aortic lesions were found in 16 of 93 (17%) black-backed jackals. Of these, four (25%) were associated with *S. lupi* larvae. Genotyping of the larvae revealed amplification of all nine loci that amplified dog-derived *S. lupi,* with the same level of polymorphism in the allele size ranges. Only 1 of 93 jackals had an esophageal nodule with concurrent *S. lupi*-induced aortic aneurysms. The single esophageal nodule found did not contain adult nematodes, nor did it communicate with the esophageal lumen. None of the jackals that were examined had macroscopically evident spondylitis, which is frequently reported in the dog.

Histopathology of the *S. lupi-*induced aortic lesions in the jackal revealed replacement of elastic and smooth muscle fibers by fibrous connective tissue. In cases where inflammation was present, the inflammatory infiltrate consisted predominantly of eosinophils. The single esophageal nodule histologically resembled the early inflammatory nodule described in dogs and consisted of fibrous connective tissue, multifocal accumulation of lymphocytes, plasma cells and rare hemosiderin-laden macrophages.

**Conclusions:**

These lesions suggest that the life cycle of *S. lupi* may not or only rarely be completed in jackals. A possible explanation might be that jackals are relatively resistant to developing significant pathology associated with *S. lupi-*infection. However, before any conclusions can be drawn, many more jackals, including those that die naturally will have to be investigated for evidence of *S. lupi* infection*.*

## Background

*Spirocerca lupi* is a spirurid nematode of *Canidae*, especially domestic dogs (*Canis familiaris*)*,* which occurs particularly in the tropical and subtropical regions of the world [4]. Dogs become infected by eating the intermediate host (i.e. coprophagous dung beetles), paratenic hosts (e.g. lizards) containing encysted larvae or through coprophagia [14, 42]. Larvae excyst in the stomach, penetrate the gastric mucosa and migrate in the wall of the gastric, gastro-epiploic and celiac arteries to reach the caudal thoracic aorta where they mature to adults. Young adult worms then migrate from the aorta to the caudal esophagus where they develop to mature worms within nodules in the esophageal submucosa and adventitia [42]. Female worms burrow through the esophageal mucosa to establish an opening to the lumen where eggs are deposited, thereby completing the life cycle [42]. Not all ingested larvae reach the aorta; some migrate to aberrant sites, including the kidney, urinary bladder wall, subcutaneous tissues, interdigital skin, trachea, mediastinum, lung and spinal cord [3, 13, 16].

Lesions pathognomonic for spirocercosis in dogs are well documented and include scarring and mineralization of the caudal thoracic aorta with aneurysm formation, spondylitis of the ventral aspect of the caudal thoracic vertebrae and the formation of caudal esophageal nodules [17]. Microscopic aortic lesions that have been reported in dogs include hemorrhage and neutrophil infiltration into vessel walls, smooth muscle and elastic fiber degeneration with replacement by collagen, and occasional foci of mineralization and heterotopic bone formation [15, 42]. Histologically, early esophageal nodules are inflammatory and collagenous. They consist of central necrotic tracts associated with worms and cell debris, surrounded by a collar of degenerate neutrophils, fewer eosinophils and peripheral collagenous stroma with foci of lymphoplasmacytic inflammation. More mature nodules are predominantly fibroblastic with multiple peripheral foci of lymphoplasmacytic inflammation. In 20% of cases the nodules proceed to sarcomatous neoplasia, namely osteosarcoma, fibrosarcoma or anaplastic sarcoma [15, 18]. Esophageal nodules have erroneously been described as granulomas in the past [3, 4], but although scattered macrophages are often present, they rarely predominate as would be expected in granulomatous inflammation [17–19, 42]. Histologically, the spirocercosis-related spondylitic lesion that occurs in close proximity to the peri-aortitis and aortic aneurysms in the caudal thoracic aorta consists of periosteal new woven bone formation perpendicular to and continuous with the underlying mature cortical bone. Scarce lymphoplasmacytic inflammation is observed in tissues adjacent to the periosteum [26].

Spirocercosis has been reported in a variety of wild carnivores, including the coyote (*Canis latrans),* wolf (*Canis lupus*), red fox (*Vulpes vulpes*), grey fox (*Urocyon cinereoargenteus*), neotropical bush dog (*Speothos venaticus*), bobcat (*Felis rufus*) and cheetah (*Acinonyx jubatus)* [4, 12, 30, 32, 35, 38]. *S. lupi-*induced aortic lesions were found in 123 of 150 (82%) coyotes, 23 of 66 (35%) bobcats, one of five (20%) grey foxes and one of two (50%) red foxes examined in West Texas from 1973 to 1977 [32]. The larvae were identified as *S. lupi* by microscopy, which has low specificity [39]*.*

Black-backed jackals are widely distributed throughout southern Africa, are especially prevalent in farming areas and are also closely associated with domestic dogs, particularly in rural and semi-rural areas [37]. The jackal diet includes a large proportion of insects (including beetles), small mammals and lizards and therefore they may represent a reservoir host for *Spirocerca* species [28, 41]. This study reports the results of a survey on 93 black-backed jackals with emphasis on the prevalence of infection, the pathology of infection and the genotype of the parasite in this species.

## Methods

### Sample population

Black-backed jackals that were routinely culled by farmers in the North West, Gauteng and Mpumalanga provinces of South Africa during the period June 2012 to February 2013 were selected for the study.

### Baseline data

Baseline data including age, sex and geographic location were recorded. Based on dentition, jackals were categorized as pups of one to six months of age, juveniles of seven to twelve months of age and adults older than one year, according to a published technique [27], together with R. Harrison-White’s personal experience from known age groups of recaptured wild jackals. This technique involves the determination of the amount of wear on the cusps and fissures (grooves) of the first two incisors. One to six month old jackals were classified as such by the presence of deciduous teeth as all of the permanent teeth are present in jackal from the age of six-months [21]. Juvenile jackals were identified by the presence of a full set of permanent teeth with the absence of any wearing of the cusps on both incisor 1 and incisor 2. Jackals older than one year showed variable wear on the cusps and fissures (grooves) of the first two incisors. The proportion of *S. lupi*-associated lesions was compared by age and sex using chi-square or Fisher exact tests in available software (Epi Info, version 6.04, CDC, Atlanta, Georgia, USA). Statistical significance was established at *P* < 0.05.

### Gross examination

Necropsies were performed on all jackals following the standard necropsy technique used by the Pathology Section, Department of Paraclinical Sciences, Faculty of Veterinary Science, University of Pretoria. The aorta, vertebrae, esophagus, stomach and intestines were examined for possible *Spirocerca-*associated lesions and other organs / tissues (including the skin, subcutis, mediastinum, kidneys, urinary bladder, spleen, liver, heart, lung and trachea) were carefully examined for signs of aberrant migration. Macroscopically visible lesions were recorded, measured and described in detail. Tissues from macroscopically visible lesions were sampled and preserved in 10% buffered formalin for microscopic evaluation. In the event of aortic pathology being present, the whole aorta and adventitial lymph nodes (*Lnn. Lumbales aortici)* (when present) were collected and placed in 10% buffered formalin. Samples of aortas from an additional six uninfected jackals were collected and fixed in 10% buffered formalin to serve as negative tissue controls to facilitate accurate interpretation of aortic pathology. The thoracic vertebrae were examined for evidence of spondylitis.

### Histopathology

Samples collected for light microscopic evaluation were wax-embedded, sectioned and stained with hematoxylin-eosin (H&E) following standard methods [5]. Processing of collected samples was done by the histotechnology laboratory of the Faculty of Veterinary Science, University of Pretoria. Light microscopic evaluation of tissue sections was done by the primary investigator using an Olympus model CX21FS1 light microscope. Aortic sections were additionally stained with Masson’s Trichrome and Verhoeff’s stain following standard methods for the detection of fibrous connective tissue and elastic fibers, respectively [5].

The severity and duration of inflammation within the sections was graded as mild, moderate or severe and as acute, subacute, chronic or chronic-active, respectively (Table 1).

**Table 1:** Grading of inflammation of histopathology tissue sections

Grading of inflammation			
Severity		Duration	
Mild	≤400 inflammatory cells in one high power field (400X magnification)	Acute	Predominance of polymorphonuclear leukocytes (neutrophils and eosinophils), accompanied by prominent inflammatory edema.
Moderate	400–900 inflammatory cells in one high power field (400X magnification)	Subacute	Polymophonuclear inflammatory infiltrate mixed with mononuclear cells (lymphocytes, macrophages and plasma cells). Edema present, but mild.
Severe	>900 inflammatory cells in one high power field (400X magnification)	Chronic	Large numbers of plasma cells, lymphocytes, macrophages and significantly fewer polymorphonuclear leukocytes, accompanied by small blood vessel proliferation and fibroblasts (granulation tissue).
		Chronic - active	Large numbers of plasma cells, lymphocytes, macrophages and polymorphonuclear leukocytes, accompanied by proliferation of small blood vessels and fibroblasts (granulation tissue).

### Genotyping of *Spirocerca* larvae

Larvae that were collected from the subintimal surface of the thoracic aorta as well as from peri-aortic nodules were submitted in 95% ethanol to the Department of Genetics, University of Pretoria, for genotyping. Previously published protocols were used for the deoxyribonucleic acid (DNA) extraction, polymerase chain reaction (PCR) amplification and sequencing [11]. Polymorphic forward primers of nine microsatellite loci for *S. lupi* from domestic dogs were labeled fluorescently. After combining the nine primer sets into a single multiplex reaction, the Quantitect Multiplex PCR kit (Qiagen) was used according to the manufacturer’s protocol. An ABI3500xl Genetic Analyser (Applied Biosystems), using GeneScan LIZ500 Internal Size Standard (Applied Biosystems) was used to separate and measure all allele sizes. By evaluating chromatograms in GeneMarker™ version 2.4.0. (SoftGenetics LLC), the genotype of each individual larva was established. Following published protocols, using the program Arlequin version 3.5.1, pairwise F_ST_ values were calculated from larvae collected from jackals and from dogs from three different provinces in South Africa [20].

## Results

A total of 51 female and 42 male jackals were examined, of which 67 originated from the North West province, 18 from Gauteng province and 8 from Mpumalanga province of the Republic of South Africa. *S. lupi*-associated aortic lesions were identified in 16 (17%) jackals and did not vary by sex (*P* = 0.965), with 9 being female (56%) and 7 being male (44%). Three of 30 juvenile jackals (10%) and 13 of 48 adult jackals (27%) had *S. lupi-*associated lesions. Thirteen pups were examined, none of which showed any *S. lupi-*associated lesions. Overall, older jackals were more likely than juvenile jackals and pups to have *S. lupi*-associated lesions (*P* = 0.018). A total of 13 larvae were recovered from four of the *S. lupi-*positive cases. A single 4-year-old jackal with *S. lupi-*induced aortic aneurysms also had an esophageal nodule that was devoid of adult nematodes. None had macroscopically evident spondylitis and no lesions suggestive of larval migration were present in the stomach, intestines or other organs.

### Gross pathology

**Fig. 1:**
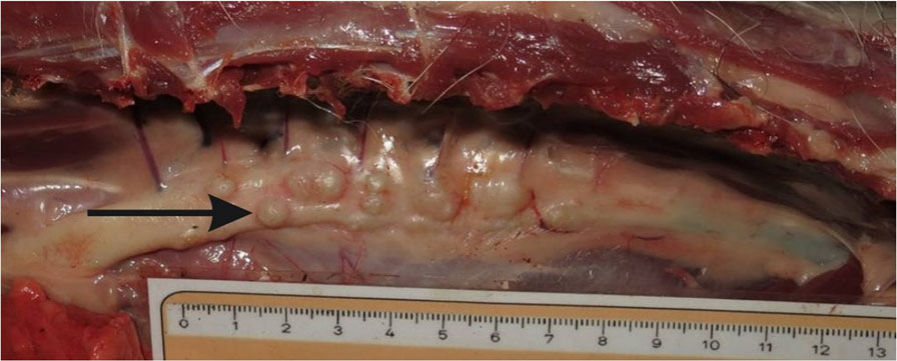
Macroscopic view of aortic aneurysms in a jackal with *S. lupi* (→)

**Fig. 2:**
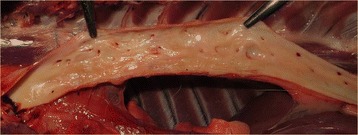
Macroscopic view of the intimal surface of aortic aneurysms in a jackal with *S. lupi*

Gross lesions in the aorta ranged from plaque-like lesions, intimal pitting consisting of a roughened, irregular intima to aortic aneurysms of variable size (Figs. 1 and 2). In two cases, small red *S. lupi* larvae were visible beneath the intimal surface. These were associated with small hemorrhages and hyperemia of the intimal surface. Older aortic lesions showed scarring and thickening of the aorta, with loss of elasticity, and aortic aneurysms were visible from the serosal aspect of the aorta. All aortic lesions were confined to the extra-cardiac, distal thoracic aorta. Observed aneurysms were mostly located in the region of thoracic vertebrae (T) T10 to T13, but ranged from T6-T13. The smallest aneurysm measured 3 mm (mm) in length, 3 mm in breadth and 1 mm in depth, whilst the largest was 12x13x10 mm. The median number of aneurysms was 3 and the range 1–6.

**Fig. 3:**
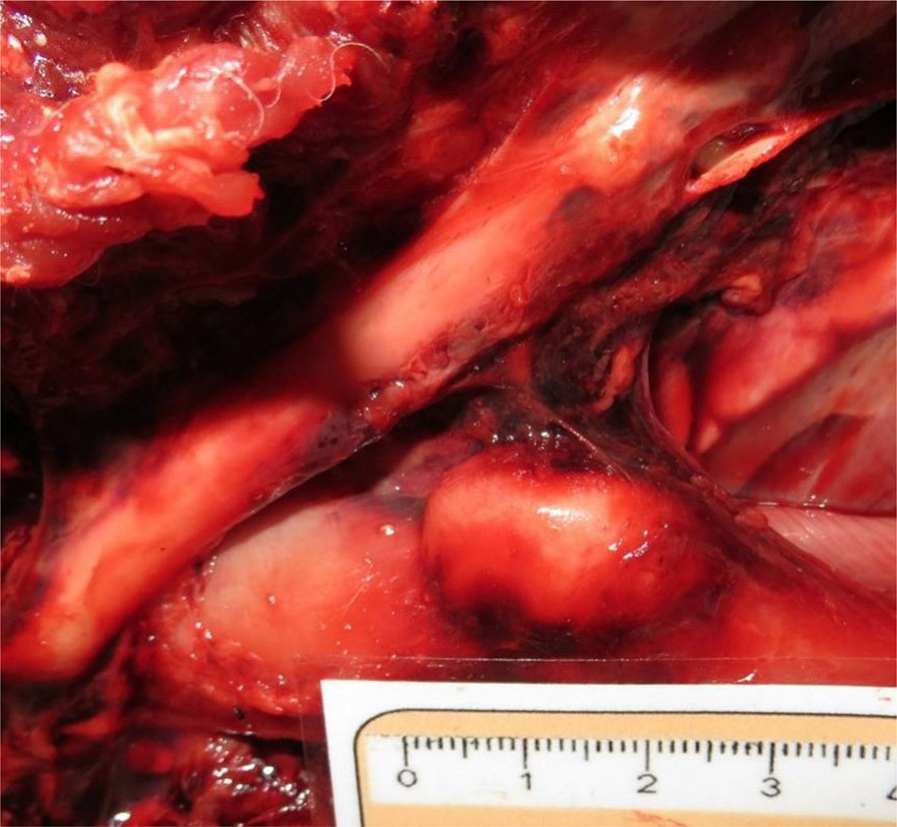
Macroscopic view of the esophageal nodule in an infected jackal

Only one esophageal nodule was present in one of the jackals examined (a 4 year-old female jackal with concurrent aortic aneurysms) (Fig. 3). The sessile, cream-colored nodule was firm and smooth to the touch and mineralization was absent. It was not ulcerated, there was no communication with the esophageal lumen, investigated using a 1 mm diameter blunt probe, and it contained no adult nematodes. The nodule was situated between the 8th and 9th thoracic vertebrae, 58 mm cranial to the diaphragm and measured 25 mm in length, 14 mm in breadth and 12 mm in depth. There was no evidence of esophagitis, esophageal dilatation proximal to the nodule or esophageal stricture formation. No other nodules were identified in that or any other jackal.

### Histopathology

**Fig. 4:**
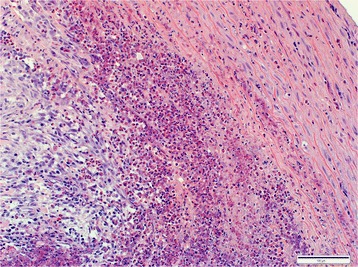
Histological transverse section of the aorta in the chronic-active inflammatory stage in an infected jackal. Note the abundance of eosinophils. Hematoxylin and eosin (HE)

**Fig. 5:**
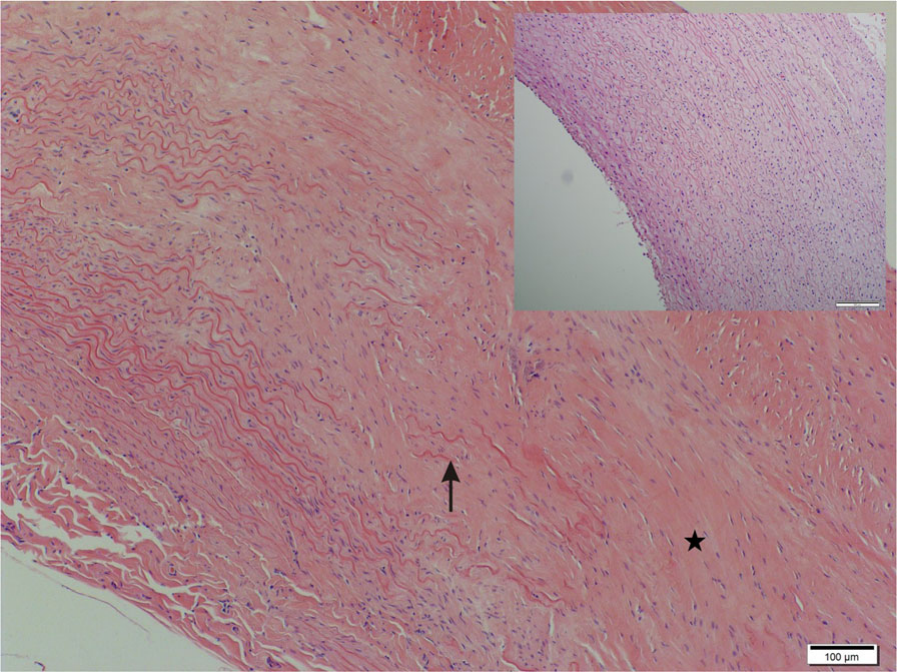
Histological transverse section of the aorta in the repair via fibrosis stage in an infected jackal. Note the elastic fiber degeneration (↑), mural fibrosis (*) and the absence of inflammatory cells. HE. *Inset:* histological transverse section of a normal aorta in a non-affected jackal at the same magnification. HE

**Fig. 6:**
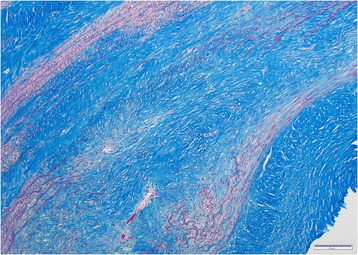
Histological transverse section of the aorta in an infected jackal. Note the extensive fibrosis (blue). Masson’s Trichrome stain

**Fig. 7:**
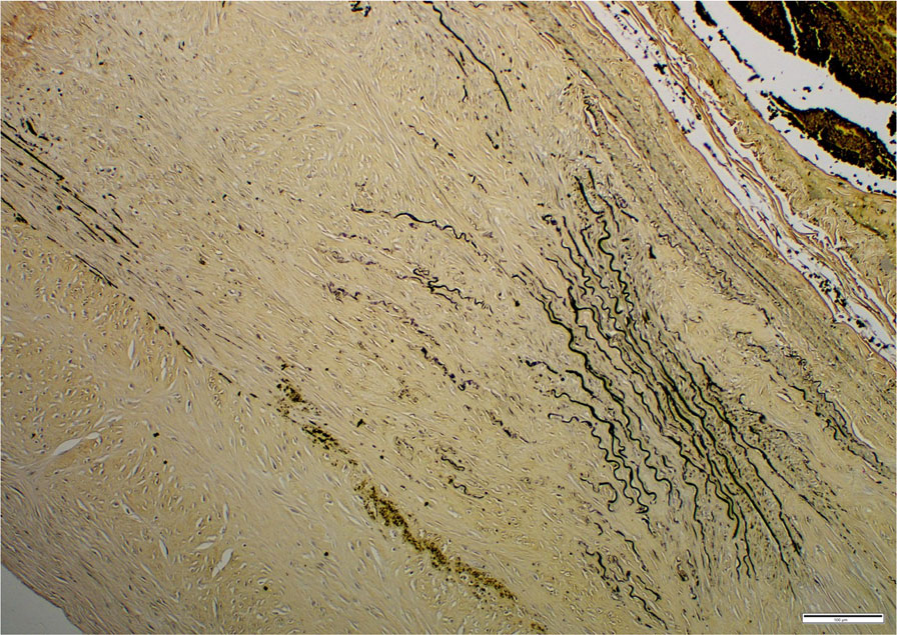
Histological transverse section of the aorta in an infected jackal. Note the elastic fiber (black) disruption and destruction. Verhoeff’s stain

Lesions in all aortas from infected jackals were either in a chronic or chronic-active stage of inflammation or there was simply evidence of repair via fibrosis with no concurrent inflammation. The chronic-active cases (Fig. 4) were characterized by large numbers of eosinophils, plasma cells, lymphocytes and fewer neutrophils, hemosiderin-laden macrophages and the occasional mast cell in the tunica adventitia (mainly) and tunica media. The inflammation was severe and predominantly focally-extensive and frequently associated with larvae. Larval migration tracts consisted of necrotic cellular debris, surrounded by the above-mentioned inflammatory cells (especially eosinophils) and copious amounts of fibrin. Also associated with the tracts were large numbers of plump fibroblasts and small caliber blood vessels with hypertrophied endothelial cells (granulation tissue). Following published descriptive techniques, larval cross sections were characterized from the periphery to the interior by a thick, smooth outer cuticle followed by a hypodermis that expanded into the pseudocelom to form two prominent stalked lateral chords, celomyarian-polymyarian musculature, moderate amounts of eosinophilic material in the pseudocelom and a large intestine lined by uninucleate cuboidal epithelial cells with a prominent tall brush border [23]. No mineralization was evident in the larval sections examined. Chronic inflammatory cases were characterized by mild to moderate multifocal inflammation within the tunica media and tunica adventitia. The inflammatory infiltrate consisted predominantly of plasma cells and fewer lymphocytes, hemosiderin-laden macrophages, eosinophils and scant mast cells. Healed aortic lesions (Fig. 5) were characterized by the absence of inflammatory cells and replacement of the elastic and smooth muscle fibers within the tunica intima, −media and -adventitia by fibrous connective tissue compared with the normal aorta (Fig. 5 inset). All 16 cases (100%), however, showed fibrosis and elastic fiber destruction, mainly within the tunica intima and –media and to some extent in the tunica adventitia, whether inflammation was present or not. Fibrosis ranged from mild to severe, multifocal to coalescing and stained blue with Masson’s Trichrome stain. Fibrosis was associated with elastic fiber destruction within the tunica intima, −media and –adventitia (Fig. 6). The internal elastic lamina of the tunica intima was disrupted and the endothelial layer was inconspicuous. The remaining elastic fibers (black with Verhoeff’s stain) (Fig. 7) were intermittently dispersed throughout the collagen bundles or were haphazardly arranged in bundles and perpendicularly orientated to the collagen. In severe cases there was complete replacement of elastic fibers within the tunica media by collagen. Eleven cases (69%) had fibroblast metaplasia with mineralization, cartilage and/or heterotopic bone formation with the occasional presence of hematopoietic cells within the tunica media.

**Fig. 8:**
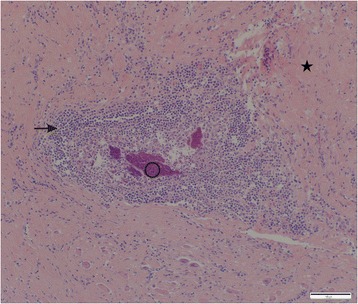
Histological section of the esophageal nodule in an infected jackal. [inflammatory cells (→), calcified necrotic debris (o), collagen (*)]. HE

Histologically the esophageal nodule was characterized by large amounts of collagen within the submucosa and extending into the tunica muscularis, with intervening mature fibrocytes and multifocal small clusters of inflammatory cells consisting predominantly of plasma cells and fewer lymphocytes, macrophages and occasional eosinophils. The inflammatory cell infiltrate usually surrounded small foci of necrotic cellular debris, which were often mineralized (Fig. 8). No nematodes or eggs were present in the sections examined.

### Genotyping

All nine loci that amplified dog-derived *S. lupi* also produced amplification in larvae that were collected from jackals. All four *S. lupi* populations (three from dogs and one from jackals) showed very small, but significant differentiation from one another. Pairwise F_ST_ values were <0.093, all *P* < 0.01 indicating a single population.

## Discussion

Our study showed that *S. lupi* is present in the black-backed jackal population in South Africa based on the microsatellite data as well as macroscopic and microscopic findings. The molecular study indicated a close relationship between the *S. lupi* found in dogs and jackals in South Africa. This was illustrated by the amplification of all nine loci in larvae collected from jackals similar to the dog-derived *S. lupi,* with the same levels of polymorphism that were observed as for dogs, where the allele size ranges of the jackal-derived samples fell within the range that was found for dog-derived samples for most loci. The nematodes found in most of the previous studies involving wild carnivores were not molecularly characterized. However, *Spirocerca* species isolated from gastric nodules in a red fox in Denmark was genetically distinct by 7 to 9% from isolates of *S. lupi* from Africa, Europe and Asia, and the authors suggested the existence of a cryptic species within *Spirocerca* [1]*.*

The limitations of this study included the short duration of the study, the relatively small number of animals with *S. lupi-*associated lesions and the possibility that samples were not representative of all jackals, since culls were not randomly selected. This should be addressed in future research. Despite these limitations however, significant data were obtained in this study, namely the genetic classification of *S. lupi* and the presence of *S. lupi-*induced lesions in 16 of 93 jackals. The fact that there was no completion of the parasite’s life cycle in any of the 16 infected jackals certainly warrants further investigation.

The *S. lupi*-associated aortic pathology present in jackals is largely similar to that described in dogs, namely intimal thickening, aneurysm formation and dystrophic mineralization as a result of elastic fiber damage within the tunica media, with eventual fibrous replacement and ossification with hematopoietic tissue formation [3, 4, 15, 26]. These characteristics were also described in wild carnivores from the northern hemisphere [32]. The most striking difference between the *S. lupi*-associated aortic pathology in dogs and jackals is the predominance of eosinophils within larval tracts in the jackals’ aortas. In the majority of dogs that have been necropsied in South Africa, the larval tracts consist predominantly of neutrophils and far fewer eosinophils [18]. However, most *S. lupi-*induced lesions are in an advanced stage when a necropsy is requested on a dog that has died of spirocercosis, whilst dogs that have died from causes other than *S*. *lupi* might have incidental and therefore early *S. lupi-*induced lesions, similar to those in jackals. Thus far there has been no systematic study of early lesions in dogs. The *S. lupi-*induced esophageal nodule found in one jackal in this study resembled that of the early inflammatory and collagenous stage in the dog, with no worms or eggs present, no communication with the esophageal lumen and no evidence of neoplastic transformation. None of the jackals examined had caudal thoracic ventral vertebral body spondylitis, which is pathognomonic for *S. lupi* in dogs [17]. In dogs, the larvae frequently show aberrant migration, for which there was no evidence in this study. Various complications following the normal and aberrant migration of larvae in the dog have been described (aortic rupture, esophageal perforation, pneumothorax, pyothorax), none of which were observed in the jackals examined.

*Spirocerca-*induced lesions in jackals differed from those described in wild carnivores in the northern hemisphere in that larval tracts in the jackal’s aortas were dominated by eosinophils and the jackal’s esophageal nodule was predominantly fibrous, whereas larval tracts and esophageal nodules in wild carnivores in the northern hemisphere were predominantly described as granulomatous, although in coyotes eosinophils featured prominently in the cellular infiltrate [32]. On the other hand, *Spirocerca*-induced lesions in the red fox (*Vulpes vulpes*) have been reported as gastric and omental nodules characterized microscopically by fibroplasia and an inflammatory infiltrate consisting of lymphocytes, plasma cells, hemosiderin-laden macrophages and fewer neutrophils and eosinophils [1, 22].

Lack of communication of esophageal nodules as observed in the jackal may represent a general trend in wild carnivores. Of 150 coyotes examined in Texas (USA), only 11 (7%) had *S. lupi-*induced esophageal nodules and in only 5 (45%) did the worms establish communication with the esophageal lumen [32]. In a red fox with *Spirocerca-*induced gastric nodules in one study, the worms did establish communication with the gastric lumen, but no *S. lupi* eggs were found in the feces using the fecal flotation egg count technique [29].

Sarcomatous transformation of the esophageal nodule and *S. lupi*-associated spondylitits of the ventral bodies of the caudal thoracic vertebrae were not observed in the jackals in this study and have not been reported in wild carnivores [32]. However, aberrant larval migration has been reported in coyotes, as well as complications of larval migration in a neotropical bush dog (*Speothos venaticus*) and in a red fox [10, 29, 35]. Further studies are needed to establish whether aberrant larval migration or complications of larval migration occur in jackals.

The apparent low pathogenicity found in jackals in this study may suggest that spirocercosis in jackals is merely an incidental finding. However, this is purely speculative as the jackals that were culled in this study were culled for reasons unrelated to illness and the timeline and burden of infection is unknown. Furthermore, unrecorded mortalities may occur in jackals suffering from more severe infestations. Numerous theories exist to explain the differences in pathologies observed in jackals, other wild carnivores (e.g. coyotes) and domestic dogs with spirocercosis, some of which are briefly examined below.

One such theory is that jackals are much older hosts to *S. lupi* and have evolved resistance over time to *S. lupi* infection, whereas dogs might be a far more recent host [33, 43]. The predominance of eosinophils in the jackals’ and coyotes’ microscopic aortic sections may support the idea that these carnivores may have evolved resistance towards *S. lupi* infection. Eosinophils are potent cells capable of combatting helminth infections and maintaining tissue homeostasis, which significantly influences the efficiency of the innate immune response [6, 36] and presumably helps to minimize the *S. lupi* burden, thus protecting the host at the expense of the parasite [33].

Another theory to consider might be that the dog’s immune system is relatively immature and therefore suboptimal for dealing with *S. lupi* infections. During the process of selection and domestication of dogs, many of the structural modifications of modern breeds are evident in changes in the rate of development [9]. These changes are dominated by pedomorphosis (the retention of juvenile morphological characteristics) [24]. It is possible that pedomorphosis has also rendered the dogs’ immune system immature.

The difference in the immune response to parasitic infection between urban dogs and jackals might also be due to jackals being scavengers [34], as are coyotes [2]. However, free-ranging or feral dogs are also efficient scavengers, especially in under-resourced communities [8]. Scavengers such as jackals, coyotes, free-ranging and feral dogs have increased exposure to a wider variety and number of microorganisms and/or toxins from decomposed carcasses and refuse [7]. Therefore, they may have evolved an improved innate immune response to infection compared to the domestic dog, which may have an inadequate innate immunity as a result of selective breeding and reduced priming of the immune system. This might explain the abundance of eosinophils in the aortic lesions of the infected jackals examined in this study. Future research should address the immune response against parasitic infections in scavenging (i.e. free-ranging and feral) dogs, versus non-scavenging (i.e. urban) dogs to further evaluate this aspect.

Finally, another aspect worthy of investigation is the composition of the intestinal microflora in jackals, free-ranging and feral dogs and urbanized domestic dogs (i.e. scavengers versus non-scavengers). Various factors such as diet, the use of antibiotics and microbial inoculation can bring about permanent or transient changes in the intestinal microflora and can involve up to 20% of the bacterial strains [25, 31]. Since the main immunological function of gut microflora is the development and maintenance of homeostasis of local and systemic immunity, the composition of the intestinal microflora should influence the immune response [25]. Whereas it is known that antibiotics have a profound effect on the composition of intestinal microflora, the consumption of highly specialized and purified commercial diets by urban dogs is bound to influence the intestinal microflora composition versus the varied diet of jackals, coyotes and scavenger dogs [31]. It seems feasible therefore that specialized diets and antibiotic therapy would impact on the immune response to *S. lupi* infection in urban dogs [31].

## Conclusion

It has been established that *S. lupi* is present in the black-backed jackal population in South Africa, but in the sample population in this study the parasite did not appear to complete its life cycle. A possible explanation for this is that jackals may have evolved resistance towards *S. lupi* infection with only moderate immune responses that minimize energetic investment as well as associated pathologies. However, due to the relatively small sample size of this study, there is a clear need to investigate *S. lupi* infestation in a far greater number of jackal. Such an investigation should include necropsies on sick jackals that die and surveys of jackals’ feces for the presence of *S. lupi* DNA [40].

## Abbreviations

DNA: Deoxyribonucleic acid
et al.: et alia (and others)
Fig.: Figure
HE: Haematoxylin and eosin
i.e.: id est. (that is)
mm: millimeter
PCR: Polymerase chain reaction
T: Thoracic vertebrae
